# Crystal structure of 7-iodo-4-oxo-4*H*-chromene-3-carbaldehyde

**DOI:** 10.1107/S2056989016016972

**Published:** 2016-11-04

**Authors:** Yoshinobu Ishikawa

**Affiliations:** a School of Pharmaceutical Sciences, University of Shizuoka, 52-1 Yada, Suruga-ku Shizuoka 422-8526, Japan

**Keywords:** crystal structure, π–π stacking, hydrogen bond, halogen bond

## Abstract

In the title compound, C_10_H_5_IO_3_, an iodinated 3-formyl­chromone derivative, the non-H atoms are essentially coplanar (r.m.s. deviation = 0.0344 Å), with the largest deviation from the least-squares plane [0.101 (3) Å] being found for the formyl O atom. In the crystal, mol­ecules are linked through stacking inter­actions [centroid–centroid distance between the benzene rings = 3.700 (3) Å] and C—H⋯O hydrogen bonds. Halogen bonds between the I atoms at 7-position and the formyl O atoms [I1⋯O3 = 3.056 (2) Å, C6—I1⋯O3 = 173.18 (8)° and I1⋯O3—C10 = 111.12 (18)°] are also formed along [110], resulting in sheets perpendicular to the *c* axis, constructed by C—H⋯O hydrogen bonds and I⋯O halogen bonds.

## Abstract 2

In the crystal of 7-iodo-4-oxo-4*H*-chromene-3-carbaldehyde, mol­ecules are linked through stacking inter­actions and C—H⋯O hydrogen bonds. Halogen bonds between the formyl O and I atoms are also formed.

## Chemical context   

3-Formyl­chromone and its derivatives show versatile biological activities such as anti-inflammatory activity (Khan *et al.*, 2010 ▸) and the inhibition of protein tyrosine phosphatase 1B (Shim *et al.*, 2005 ▸), thymidine phospho­rylase (Khan *et al.*, 2009 ▸), carbonic anhydrase (Ekinci *et al.*, 2012 ▸), and metallo-β-lactamase (Christopeit *et al.*, 2016 ▸). Inter­estingly, 6,8-di­chloro- and 6,8-di­bromo-3-formyl­chromones possess potent urease inhibitory activity, whereas 6-fluoro-, 6-chloro- and 6-bromo-3-formyl­chromones exhibit no ability to inhibit urease (Kawase *et al.*, 2007 ▸). Thus, the position of halogen atoms on the chromone ring should be associated with the urease inhibitory activity.
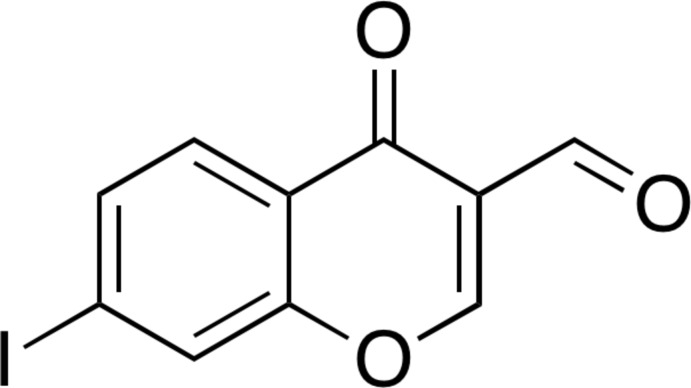


We have previously reported the crystal structures of 6,8-di­chloro-4-oxochromene-3-carbaldehyde (6,8-di­chloro-3-formyl­chromone; Ishikawa & Motohashi, 2013 ▸) and 6,8-di­bromo-4-oxo-4*H*-chromene-3-carbaldehyde (6,8-di­bromo-3-formyl­chromone; Ishikawa, 2014*a* ▸). In these crystals, halogen bonds are observed between the formyl oxygen atoms and the halogen atoms at the 8-position. Halogen bonding is defined as a net attractive inter­action between an electrophilic region of a halogen atom in a mol­ecule and a nucleophilic region of an atom in a mol­ecule, and is characterized by a shorter contact between the two atoms. Halogen bonding has attracted much attention in medicinal chemistry, chemical biology, supra­molecular chemistry and crystal engineering (Scholfield *et al.*, 2013 ▸; Wilcken *et al.*, 2013 ▸; Persch *et al.*, 2015 ▸; Cavallo *et al.*, 2016 ▸).

As part of an investigation of halogenated 3-formyl­chromones relevant to urease inhibitory activity and halogen bonding, I herein report the crystal structure of 7-iodo-4-oxo-4*H*-chromene-3-carbaldehyde (7-iodo-3-formyl­chromone). The main objective of this study is to reveal the inter­action mode of the iodine substituent at the 7-position of the chromone ring in the solid state.

## Structure commentary   

**Figure 1:**
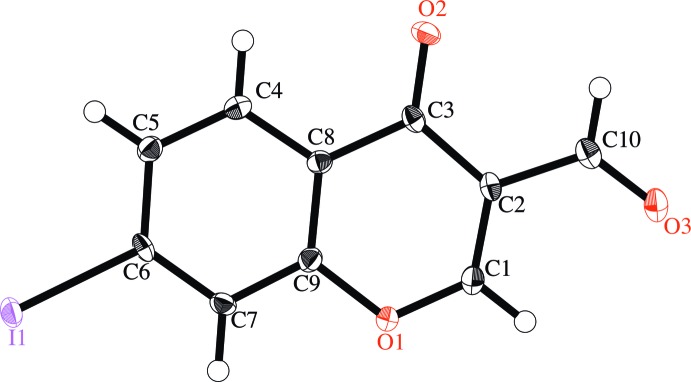
The mol­ecular structure of the title compound, with displacement ellipsoids drawn at the 50% probability level. H atoms are shown as small spheres of arbitrary radius.

The mean deviation of the least-square planes for the non-hydrogen atoms is 0.0344 Å, and the largest deviation is 0.101 (3) Å for O3, indicating that these atoms are essentially coplanar (Fig. 1 ▸). All bond distances and angles are within their expected ranges.

## Supra­molecular features   

**Figure 2:**
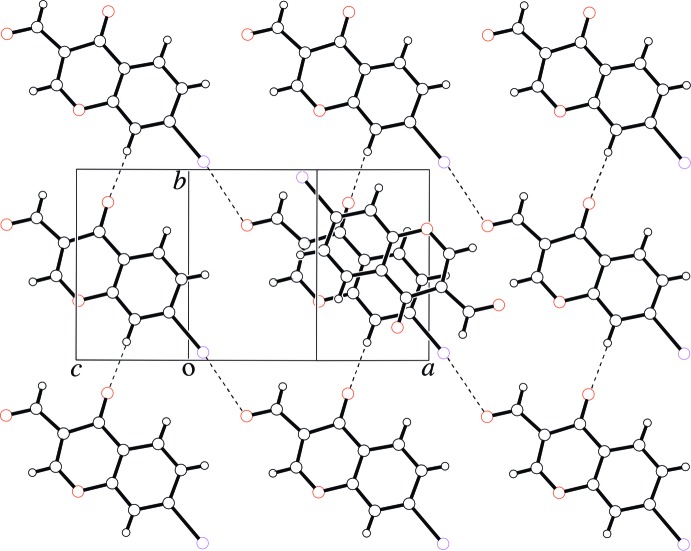
A packing view of the title compound. C—H⋯O hydrogen bonds and I⋯O halogen bonds are represented as dashed lines.

In the crystal, the mol­ecules are linked through π–π stacking inter­actions between inversion-symmetry-equivalent^i^ mol­ecules [centroid–centroid distance between the benzene rings of the 4*H*-chromene units = 3.700 (3) Å; symmetry code: (i) −*x*, −*y*, −*z*], and through C—H⋯O hydrogen bonds (Table 1 ▸) that involve the C7/O2 atoms. In particular, significant shorter contacts are observed between the iodine atoms and the formyl oxygen atoms of translation-symmetry equivalent^ii^ mol­ecules [I1⋯O3 = 3.056 (2) Å, C6—I1⋯O3 = 173.18 (8)°, I1⋯O3—C10 = 111.12 (18)°; symmetry code: (ii) *x* + 1, *y* − 1, *z*] along [1 1 0], resulting in sheets perpendicular to the *c*-axis, constructed by C—H⋯O hydrogen bonds and I⋯O halogen bonds (Fig. 2 ▸).

## Database survey   

**Figure 3:**
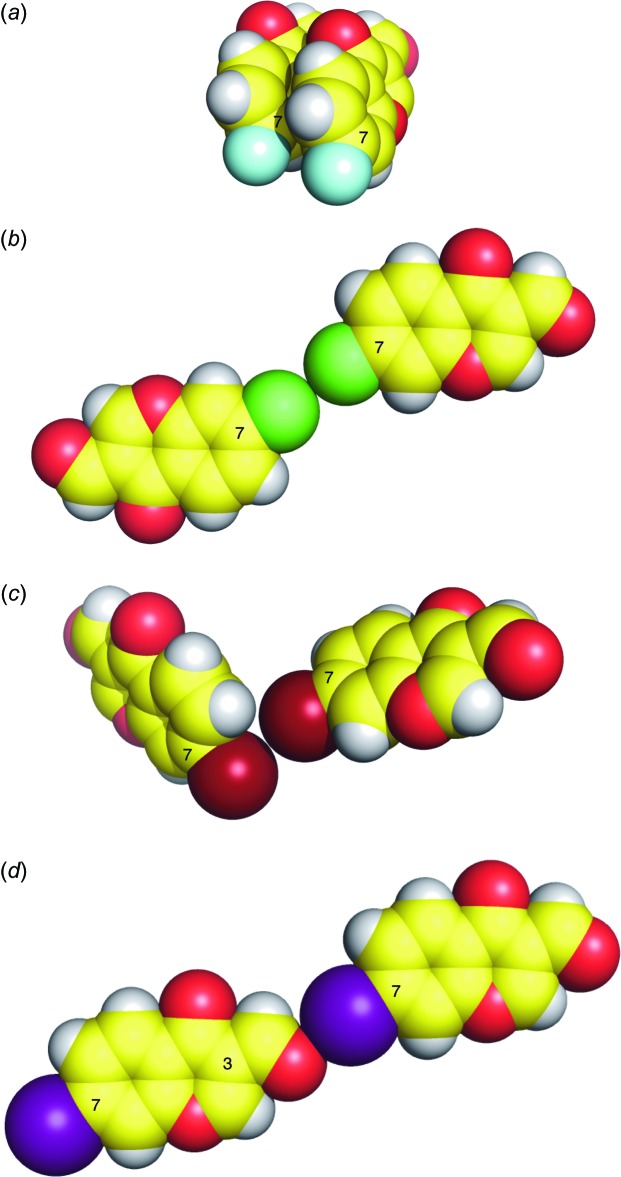
Sphere models of the crystal structures of (*a*) 7-fluoro-4-oxo-4*H*-chromene-3-carbaldehyde (Asad *et al.*, 2011 ▸), (*b*) 7-chloro-4-oxo-4*H*-chromene-3-carbaldehyde (Ishikawa, 2014*b* ▸), (*c*) 7-bromo-4-oxo-4*H*-chromene-3-carbaldehyde (Ishikawa, 2014*c* ▸) and (*d*) the title compound (this work).

A search of WebCSD (Version 1.1.2, last update Oct 2016; Groom *et al.*, 2014 ▸) for 7-halogeno-3-formyl­chromones gave the following three hits: 7-fluoro- (Asad *et al.*, 2011 ▸), 7-chloro- (Ishikawa, 2014*b* ▸), and 7-bromo-3-formyl­chromone (Ishikawa, 2014*c* ▸). In 7-fluoro-3-formyl­chromone, no contact around the fluorine atom is seen (Fig. 3 ▸ *a*). In the crystals of 7-chloro- and 7-bromo-3-formyl­chromones, type I and type II halogen⋯halogen contacts are found, respectively (Fig. 3 ▸ *b* and 3*c*), and these halogen⋯halogen contacts are commonly found for Cl and Br atoms (Mukherjee *et al.*, 2014 ▸). It should be noted that shorter contacts between oxygen atoms and halogen atoms are observed in 7-iodo-3-formyl­chromone (this work, Fig. 3 ▸ *d*), but not in 7-fluoro-, 7-chloro-, and 7-bromo-3-formyl­chromones. This is in agreement with an assumption that the iodine atom should have the largest σ-hole (Clark *et al.*, 2007 ▸) among the halogen atoms in 7-halogeno-3-formyl­chromones. These findings should be helpful in understanding the inter­action of halogenated 3-formyl­chromones with urease, and is thus valuable for rational drug design.

## Synthesis and crystallization   

2′-Hy­droxy-4′-iodo­aceto­phenone was prepared from 3-acetoxy­iodo­benzene by a Fries rearrangement reaction. To a solution of 2′-hy­droxy-4′-iodo­aceto­phenone (4.4 mmol) in *N*,*N*-di­methyl­formamide (10 ml) was added dropwise POCl_3_ (13.2 mmol) at 273 K. After the mixture was stirred for 14 h at room temperature, water (100 ml) was added. The precipitates were collected, washed with water, and dried *in vacuo* at 333 K (yield 86%). ^1^H NMR (400 MHz, CDCl_3_): δ 7.84 (dd, 1H, *J* = 8.8 and 1.5 Hz), 7.96 (d, 1H, *J* = 1.5 Hz), 7.99 (d, 1H, *J* = 8.3 Hz), 8.49 (s, 1H), 10.36 (s, 1H). Single crystals suitable for X-ray diffraction were obtained by slow evaporation of a 1,2-di­chloro­ethane solution of the title compound at room temperature.

## Refinement   

Crystal data, data collection and structure refinement details are summarized in Table 2 ▸. The C-bound hydrogen atoms were placed in geometrical positions and refined using a riding model [C—H = 0.95 Å and *U*_iso_(H) = 1.2*U*_eq_(C)].

## Supplementary Material

Crystal structure: contains datablock(s) global, I. DOI: 10.1107/S2056989016016972/zl2682sup1.cif

Structure factors: contains datablock(s) I. DOI: 10.1107/S2056989016016972/zl2682Isup2.hkl

Supporting information file. DOI: 10.1107/S2056989016016972/zl2682Isup3.cml

CCDC reference: 1511202

Additional supporting information: crystallographic information; 3D view; checkCIF report

## Figures and Tables

**Table 1:** Hydrogen-bond geometry (Å, °)

*D*—H⋯*A*	*D*—H	H⋯*A*	*D*⋯*A*	*D*—H⋯*A*
C7—H4⋯O2^i^	0.95	2.35	3.208 (4)	150 (1)

Symmetry code: (i) 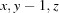.

**Table 2:** Experimental details

Crystal data	
Chemical formula	C_10_H_5_IO_3_
*M*_r_	300.05
Crystal system, space group	Monoclinic, *P*2_1_/*n*
Temperature (K)	100
*a*, *b*, *c* (Å)	9.572 (4), 7.533 (4), 13.095 (9)
β (°)	103.06 (4)
*V* (Å^3^)	919.8 (9)
*Z*	4
Radiation type	Mo *K*α
μ (mm^−1^)	3.46
Crystal size (mm)	0.25 × 0.18 × 0.15

Data collection	
Diffractometer	Rigaku AFC7R
Absorption correction	ψ scan (North *et al.*, 1968 ▸)
*T*_min_, *T*_max_	0.528, 0.595
No. of measured, independent and observed [*F* ^2^ > 2.0σ(*F* ^2^)] reflections	2538, 2119, 1916
*R*_int_	0.017
(sin θ/λ)_max_ (Å^−1^)	0.650

Refinement	
*R*[*F* ^2^ > 2σ(*F* ^2^)], *wR*(*F* ^2^), *S*	0.021, 0.053, 1.06
No. of reflections	2119
No. of parameters	128
H-atom treatment	H-atom parameters constrained
Δρ_max_, Δρ_min_ (e Å^−3^)	0.56, −0.60

Computer programs: *WinAFC* (Rigaku, 1999 ▸), *SIR2011* (Burla *et al.*, 2012 ▸), *SHELXL2014* (Sheldrick, 2015 ▸) and *CrystalStructure* (Rigaku, 2015 ▸).

## supplementary crystallographic information

### Crystal data

**Table:**

C_10_H_5_IO_3_	*F*(000) = 568.00
*M**_r_* = 300.05	*D*_x_ = 2.167 Mg m^−^^3^
Monoclinic, *P*2_1_/*n*	Mo *K*α radiation, λ = 0.71069 Å
*a* = 9.572 (4) Å	Cell parameters from 25 reflections
*b* = 7.533 (4) Å	θ = 15.1–17.1°
*c* = 13.095 (9) Å	µ = 3.46 mm^−^^1^
β = 103.06 (4)°	*T* = 100 K
*V* = 919.8 (9) Å^3^	Prismatic, yellow
*Z* = 4	0.25 × 0.18 × 0.15 mm

### Data collection

**Table:**

Rigaku AFC7R diffractometer	*R*_int_ = 0.017
ω–2θ scans	θ_max_ = 27.5°, θ_min_ = 3.0°
Absorption correction: ψ scan (North *et al.*, 1968)	*h* = −12→12
*T*_min_ = 0.528, *T*_max_ = 0.595	*k* = −9→0
2538 measured reflections	*l* = −9→17
2119 independent reflections	3 standard reflections every 150 reflections
1916 reflections with *F*^2^ > 2.0σ(*F*^2^)	intensity decay: −0.3%

### Refinement

**Table:**

Refinement on *F*^2^	Hydrogen site location: inferred from neighbouring sites
*R*[*F*^2^ > 2σ(*F*^2^)] = 0.021	H-atom parameters constrained
*wR*(*F*^2^) = 0.053	*w* = 1/[σ^2^(*F*_o_^2^) + (0.0244*P*)^2^ + 1.8229*P*] where *P* = (*F*_o_^2^ + 2*F*_c_^2^)/3
*S* = 1.06	(Δ/σ)_max_ = 0.001
2119 reflections	Δρ_max_ = 0.56 e Å^−^^3^
128 parameters	Δρ_min_ = −0.60 e Å^−^^3^
0 restraints	Extinction correction: SHELXL2014 (Sheldrick, 2015)
Primary atom site location: structure-invariant direct methods	Extinction coefficient: 0.0024 (4)
Secondary atom site location: difference Fourier map	

### Special details

**Table:**

Geometry. All esds (except the esd in the dihedral angle between two l.s. planes) are estimated using the full covariance matrix. The cell esds are taken into account individually in the estimation of esds in distances, angles and torsion angles; correlations between esds in cell parameters are only used when they are defined by crystal symmetry. An approximate (isotropic) treatment of cell esds is used for estimating esds involving l.s. planes.
Refinement. Refinement was performed using all reflections. The weighted *R*-factor (*wR*) and goodness of fit (*S*) are based on *F*^2^. *R*-factor (gt) are based on *F*. The threshold expression of *F*^2^ > 2.0 *σ*(*F*^2^) is used only for calculating *R*-factor (gt).

### Fractional atomic coordinates and isotropic or equivalent isotropic displacement parameters (Å^2^)

**Table:**

	*x*	*y*	*z*	*U*_iso_*/*U*_eq_	
I1	0.24361 (2)	0.04012 (2)	0.39087 (2)	0.01629 (8)	
O1	−0.2715 (2)	0.3092 (3)	0.39728 (15)	0.0152 (4)	
O2	−0.1775 (2)	0.8253 (3)	0.33965 (16)	0.0172 (4)	
O3	−0.5743 (2)	0.7067 (3)	0.39144 (17)	0.0209 (4)	
C1	−0.3676 (3)	0.4391 (4)	0.3915 (2)	0.0160 (5)	
H1	−0.4603	0.4076	0.4004	0.019*	
C2	−0.3435 (3)	0.6125 (4)	0.3740 (2)	0.0135 (5)	
C3	−0.2044 (3)	0.6703 (4)	0.3577 (2)	0.0128 (5)	
C4	0.0421 (3)	0.5595 (4)	0.3565 (2)	0.0143 (5)	
H2	0.0697	0.6769	0.3434	0.017*	
C5	0.1408 (3)	0.4232 (4)	0.3655 (2)	0.0144 (5)	
H3	0.2365	0.4468	0.3604	0.017*	
C6	0.0979 (3)	0.2499 (4)	0.3822 (2)	0.0133 (5)	
C7	−0.0402 (3)	0.2124 (4)	0.3922 (2)	0.0135 (5)	
H4	−0.0685	0.0945	0.4039	0.016*	
C8	−0.0982 (3)	0.5267 (4)	0.3665 (2)	0.0123 (5)	
C9	−0.1358 (3)	0.3530 (4)	0.3847 (2)	0.0132 (5)	
C10	−0.4587 (3)	0.7427 (4)	0.3717 (2)	0.0162 (5)	
H5	−0.4422	0.8619	0.3538	0.019*	

### Atomic displacement parameters (Å^2^)

**Table:**

	*U*^11^	*U*^22^	*U*^33^	*U*^12^	*U*^13^	*U*^23^
I1	0.01410 (10)	0.01533 (11)	0.01997 (11)	0.00472 (7)	0.00501 (7)	−0.00105 (7)
O1	0.0119 (9)	0.0131 (9)	0.0217 (10)	0.0010 (7)	0.0064 (8)	0.0019 (8)
O2	0.0186 (10)	0.0101 (9)	0.0221 (10)	−0.0001 (8)	0.0029 (8)	0.0011 (8)
O3	0.0155 (10)	0.0215 (11)	0.0263 (11)	0.0054 (8)	0.0064 (8)	0.0012 (9)
C1	0.0120 (12)	0.0173 (14)	0.0190 (13)	0.0022 (10)	0.0042 (10)	0.0010 (11)
C2	0.0119 (12)	0.0149 (13)	0.0135 (12)	0.0041 (10)	0.0020 (10)	0.0009 (11)
C3	0.0132 (12)	0.0126 (12)	0.0115 (12)	0.0031 (10)	0.0001 (9)	−0.0006 (10)
C4	0.0139 (12)	0.0140 (13)	0.0149 (12)	−0.0029 (10)	0.0029 (10)	−0.0004 (10)
C5	0.0125 (12)	0.0151 (14)	0.0156 (13)	−0.0014 (10)	0.0033 (10)	−0.0017 (10)
C6	0.0154 (12)	0.0111 (12)	0.0137 (12)	0.0051 (10)	0.0035 (10)	−0.0025 (10)
C7	0.0154 (12)	0.0093 (12)	0.0154 (12)	−0.0005 (10)	0.0027 (10)	−0.0014 (10)
C8	0.0129 (12)	0.0119 (12)	0.0121 (12)	−0.0013 (10)	0.0026 (9)	−0.0010 (10)
C9	0.0121 (12)	0.0142 (13)	0.0140 (12)	−0.0008 (10)	0.0044 (10)	0.0001 (10)
C10	0.0164 (13)	0.0156 (13)	0.0153 (13)	0.0040 (11)	0.0008 (10)	−0.0012 (11)

### Geometric parameters (Å, º)

**Table:**

I1—C6	2.094 (3)	C4—C5	1.383 (4)
O1—C1	1.334 (3)	C4—C8	1.400 (4)
O1—C9	1.386 (3)	C4—H2	0.9500
O2—C3	1.230 (3)	C5—C6	1.401 (4)
O3—C10	1.222 (4)	C5—H3	0.9500
C1—C2	1.355 (4)	C6—C7	1.387 (4)
C1—H1	0.9500	C7—C9	1.389 (4)
C2—C3	1.462 (4)	C7—H4	0.9500
C2—C10	1.471 (4)	C8—C9	1.392 (4)
C3—C8	1.471 (4)	C10—H5	0.9500
			
C1—O1—C9	118.2 (2)	C7—C6—C5	121.6 (2)
O1—C1—C2	125.1 (3)	C7—C6—I1	118.6 (2)
O1—C1—H1	117.4	C5—C6—I1	119.8 (2)
C2—C1—H1	117.4	C6—C7—C9	117.7 (3)
C1—C2—C3	120.5 (2)	C6—C7—H4	121.2
C1—C2—C10	119.4 (3)	C9—C7—H4	121.2
C3—C2—C10	120.1 (3)	C9—C8—C4	118.2 (2)
O2—C3—C2	123.2 (3)	C9—C8—C3	120.2 (2)
O2—C3—C8	122.8 (2)	C4—C8—C3	121.5 (3)
C2—C3—C8	113.9 (2)	O1—C9—C7	115.5 (2)
C5—C4—C8	120.8 (3)	O1—C9—C8	122.0 (2)
C5—C4—H2	119.6	C7—C9—C8	122.5 (2)
C8—C4—H2	119.6	O3—C10—C2	123.9 (3)
C4—C5—C6	119.1 (3)	O3—C10—H5	118.0
C4—C5—H3	120.5	C2—C10—H5	118.0
C6—C5—H3	120.5		
			
C9—O1—C1—C2	0.1 (4)	O2—C3—C8—C9	−178.5 (3)
O1—C1—C2—C3	1.1 (4)	C2—C3—C8—C9	2.4 (4)
O1—C1—C2—C10	−178.8 (2)	O2—C3—C8—C4	1.2 (4)
C1—C2—C3—O2	178.6 (3)	C2—C3—C8—C4	−177.9 (2)
C10—C2—C3—O2	−1.5 (4)	C1—O1—C9—C7	179.6 (2)
C1—C2—C3—C8	−2.3 (4)	C1—O1—C9—C8	0.0 (4)
C10—C2—C3—C8	177.6 (2)	C6—C7—C9—O1	−178.8 (2)
C8—C4—C5—C6	1.6 (4)	C6—C7—C9—C8	0.7 (4)
C4—C5—C6—C7	−1.5 (4)	C4—C8—C9—O1	179.0 (2)
C4—C5—C6—I1	177.9 (2)	C3—C8—C9—O1	−1.4 (4)
C5—C6—C7—C9	0.3 (4)	C4—C8—C9—C7	−0.6 (4)
I1—C6—C7—C9	−179.1 (2)	C3—C8—C9—C7	179.1 (2)
C5—C4—C8—C9	−0.6 (4)	C1—C2—C10—O3	4.9 (4)
C5—C4—C8—C3	179.7 (2)	C3—C2—C10—O3	−175.0 (3)

### Hydrogen-bond geometry (Å, º)

**Table:**

*D*—H···*A*	*D*—H	H···*A*	*D*···*A*	*D*—H···*A*
C7—H4···O2^i^	0.95	2.35	3.208 (4)	150 (1)

Symmetry code: (i) *x*, *y*−1, *z*.
